# Shift from slow- to fast-water habitats accelerates lineage and phenotype evolution in a clade of Neotropical suckermouth catfishes (Loricariidae: Hypoptopomatinae)

**DOI:** 10.1371/journal.pone.0178240

**Published:** 2017-06-07

**Authors:** Fábio F. Roxo, Nathan K. Lujan, Victor A. Tagliacollo, Brandon T. Waltz, Gabriel S. C. Silva, Claudio Oliveira, James S. Albert

**Affiliations:** 1 Laboratório de Biologia e Genética de Peixes, Departamento de Morfologia, Universidade Estadual Paulista, UNESP, Botucatu, SP, Brazil; 2 Department of Biology, University of Toronto Scarborough, Toronto, ON, Canada; 3 Programa Ciências do Ambiente (CIAMB), Universidade Federal do Tocantins, UFT, Palmas, TO, Brazil; 4 Department of Biology, University of Louisiana at Lafayette, Lafayette, LA, United States of America; Museum National d'Histoire Naturelle, FRANCE

## Abstract

Identifying habitat characteristics that accelerate organismal evolution is essential to understanding both the origins of life on Earth and the ecosystem properties that are most critical to maintaining life into the future. Searching for these characteristics on a large scale has only recently become possible via advances in phylogenetic reconstruction, time-calibration, and comparative analyses. In this study, we combine these tools with habitat and phenotype data for 105 species in a clade of Neotropical suckermouth catfishes commonly known as cascudinhos. Our goal was to determine whether riverine mesohabitats defined by different flow rates (i.e., pools vs. rapids) and substrates (plants vs. rocks) have affected rates of cascudinho cladogenesis and morphological diversification. In contrast to predictions based on general theory related to life in fast-flowing, rocky riverine habitats, Neoplecostomini lineages associated with these habitats exhibited increased body size, head shape diversity, and lineage and phenotype diversification rates. These findings are consistent with a growing understanding of river rapids as incubators of biological diversification and specialization. They also highlight the urgent need to conserve rapids habitats throughout the major rivers of the world.

## Introduction

A growing body of research indicates that colonizing novel habitats can dramatically alter rates of evolutionary diversification. Habitat-mediated shifts in rates of speciation and morphological diversification have been documented in a wide range of organisms. Examples include aquatic amphipods [1], dragon lizards [2], and various fishes (e.g. wrasses, [3]; sea catfishes, [4]; pufferfishes, [5]; silversides, [6]; minnows, [7]). Habitats implicated in these rate shifts span broad spatial scales, from biomes (e.g. marine vs. freshwater; [1, 4–6] and ecosystems (e.g. open ocean vs. coral reefs; [5]), to habitats that are regularly interspersed at relatively small spatial scales (e.g., benthic vs. pelagic stream habitats, [7]; arboreal vs. terrestrial habitats, [2]).

Natural experiments in which clades diversify significantly more quickly or slowly after colonizing new environments are uncommon. These instances are of broad significance, though, because they yield insights into both the drivers of evolutionary disparity and the fundamental ecological differences between habitats occupied by descendent lineages. Habitats that are novel to an evolutionary lineage and correlate with accelerated diversification, for example, may be inferred to have had reduced competition and/or greater ecological opportunity than the lineage’s ancestral habitats at the time of colonization [1, 4, 6]. Conversely, habitats that correlate with reduced diversification may be inferred to have had ecological niches that were already saturated at the time of colonization (ibid.).

The robustness of such ecological inferences depends on both the number of parallel habitat transitions within a given lineage, and the degree to which the historical ecological state of each habitat can be corroborated by additional data (e.g. modern species richness; [4]). Although the competitive context and resource diversity of a habitat are the principal or only driving factors invoked by many studies of habitat-mediated evolutionary rate shifts, other possible drivers of diversification include habitat patchiness and connectivity over various spatial scales [8], and the complexity and selective strength of alternative adaptive landscapes [9–11]. Moreover, different lineages at different times may differ in their preadaptations [12] to a given physical habitat or competitive context and/or their evolutionary plasticity [13] or modularity [14], which may affect their capacity to diversify within new environments. Thus, ambiguity regarding mechanisms, as well as stochastic effects [15], can inhibit robust generalizations about a habitat’s influence on lineage diversification.

Despite these caveats, studies of habitat-mediated evolutionary rate shifts provide valuable historical tests of eco-evolutionary hypotheses. In this study, we examine the differential effects of water velocity on rates of diversification in a clade of Neotropical river fishes commonly known as cascudinhos (Loricariidae: Hypoptopomatinae), which vary in their preference for fast- vs. slow-flowing riverine habitats. Fast-flowing waters in streams and rivers exhibit phylogenetically diverse fish assemblages with remarkable morphological convergences on a similar array of phenotypic specializations, such as dorsoventral depression, attachment organs, and surface-scraping jaws [16, 17].

Although morphological convergence clearly illustrates the evolutionary specializations that frequently occur in rapids habitats [17], the long-term effect that these habitats can have on evolutionary diversification, particularly compared with adjacent slower-flowing habitats, remains poorly known. We hypothesize that, because of the apparently similar and strong selection pressures that drive morphological convergence in rapids habitats, lineages specializing in these habitats would exhibit a reduction in both overall morphological diversity and diversification rates. In contrast, the deeper, more three-dimensional and complexly-structured habitat of slow-flowing habitats should correlate with higher overall morphological diversity (e.g. [18]) and diversification rates.

Swiftly-flowing lotic riverine habitats are also associated with smaller body sizes [17, 19, 20] and greater philopatry (i.e., smaller geographic range sizes) in fishes–the latter putatively because of the greater risk that long-distance movement might lead to dislodgement and exportation from a fast-flowing environment [21]. Smaller body sizes and geographic range sizes are also both expected to correlate with increased rates of speciation [21–24]. The individually small size and highly disjunct distribution of rapids within river networks would further contribute to increased speciation rates even in the absence of increase morphological diversification rates.

We test these hypotheses by generating a densely sampled, time-calibrated molecular phylogeny for the cascudinho subfamily Hypoptopomatinae, which comprises three monophyletic tribes that vary in habitat preference: the riffle- and pool-dwelling Hypoptopomatini and Otothyrini, and the rapids-dwelling Neoplecostomini [25]. We then use this phylogeny in combination with habitat and phenotype data to test for correlation between a lineage’s occupation of fast- vs. slow-flowing habitats and its rates of speciation and diversification in head shape and maximum body size (MBS).

## Material and methods

### Taxon sampling and phylogenetic inference

All comparative analyses were performed using the time-calibrated phylogenetic hypothesis of Roxo et al. [25]. This phylogeny was inferred from partial DNA sequences of three mitochondrial loci (16S rRNA, COI, Cytb) and one nuclear locus (F-reticulon 4; 4,500 base pairs total), and included 105 species representing approximately 50% of all Hypoptopomatinae species, with every tribe, genus and major lineage represented by at least one species (see S1 Table for identities, localities and catalog numbers of all tissue vouchers). Throughout this paper, we follow the taxonomic scheme of Lujan et al. [26] in which Hypoptopomatinae is a monophyletic subfamily containing the respectively monophyletic tribes Hypoptopomatini, Otothyrini and Neoplecostomini (vs. alternative taxonomic frameworks [25, 27] in which these last three clades were treated as subfamilies).

### Ethics statement

All fishes of the present study were collected under a permanent scientific collection license in the name of Dr. Claudio Oliveira (SISBIO), in accordance with each country’s laws. Furthermore, our laboratory (Laboratório de Biologia e Genética de Peixes) has special federal permission to keep fixed animals in formalin and alcohol preserved tissues from a public collection under our care. We collected and euthanized fishes in accordance with recommendations of the Comissão de Ética na Experimentação Animal (CEEA; protocol number 388), the institutional animal care and use committee of the Universidade Estadual Paulista. Specimens were euthanized with an overdose of benzocaine, after which a piece of muscle tissue was extracted from the right side of the body and preserved in 95% ethanol. Voucher specimens were then fixed in 10% formalin for two weeks, then transferred to 70% ethanol for permanent storage.

### Time calibration

We calibrated the phylogeny of Roxo et al. [25] using an uncorrelated relaxed molecular clock in the program BEAST v1.6.2 [28]. The partitioning scheme and nucleotide substitution models for our BEAST analysis were determined using the software PartitionFinder v1.1.1 ([29]; S3 and S4 Tables in [25]). Two fossil calibrations were used to constrain divergence times throughout the phylogenetic tree: The first calibration was implemented as a normally distributed prior offset to 125 million years ago (Ma) with a standard deviation of 15 (search performed in 2.5% of upper and lower quantiles of 95.6–154.4 million years; My), which matches current estimates of a Lower Cretaceous (145–100 Ma) origin of the catfish order Siluriformes [30–32]. The second calibration was implemented using a lognormal prior mean offset to 55 Ma with a standard deviation of 1 (search performed in 2.5% of upper and lower quantiles of 59.7–291.8 My) for the origin of the genus *Corydoras* lineage. We used a birth–death model for speciation likelihood, which specifically models extinction throughout the phylogeny, and used a starting tree optimized using maximum likelihood (ML). The BEAST analysis was run for 100 million generations with tree space sampled every 1,000th generation. Stationarity and sufficient mixing of parameters (ESS >200) were evaluated using Tracer v1.5 [33], and a consensus tree was assembled using TreeAnnotator v1.6.2 [34].

### Ancestral habitat estimation

Habitat data were obtained from original descriptions of each species included in our analysis and from the personal field experience of authors FFR and VAT, who collected most of the specimens examined in this study (S2 Table). We estimated historical rates of habitat evolution using the make.simmap function in the R package phytools v0.3–10 [35] and the software SIMMAP v1.5 [36]. For the SIMMAP analysis, we generated a presence/absence matrix consisting of all species assigned to one or more of three habitat types: slow-flow/plants, fast-flow/plants, fast-flow/rocks (classifications follow [37]). The resulting matrix was run through 1,000 stochastic character map simulations. We also evaluated the fit of equal-rates (ER), symmetric (SYM), all-rates-different (ARD), and meristic macroevolutionary models to our data using the fitDiscrete function for discrete data in the R package geiger [38], which ranks models according to the Akaike information criterion (AICc; [39]).

### Speciation rate estimation

To minimize incomplete sampling biases, we accounted for all species known to be missing from our speciation analyses (see ‘Perc’ in S2 Table for the percent of sampled species of each lineage and ‘Gen-Div’ for the division of lineages in the ingroup phylogeny). To estimate rates of speciation and extinction across the subfamily Hypoptopomatinae, we used BAMM v2.1.0 [40]. BAMM assumes that speciation and extinction are heterogeneously distributed throughout a phylogeny and uses a reversible jump Markov chain Monte Carlo algorithm to explore the universe of candidate cladogenesis models [40–42]. The analysis was conducted with two chains running simultaneously for a total of five million generations. We sampled tree space every 1,000th generation and checked for MCMC convergence by plotting the log-likelihood trace using the R package BAMMtools [43], with burnin set to 0.5 (i.e. first half of all samples discarded). To account for the effects of phylogenetic uncertainty on our analyses, we conducted BAMM analyses of species diversification across 2,500 trees sampled from the posterior distribution of topologies and branch lengths. To visualize the evolutionary rate dynamics from BAMM output results we also used BAMMtools.

To specifically test the hypothesis that Neoplecostomini underwent an increase in speciation rate relative to other Hypoptopomatinae lineages, we used the MEDUSA implementation [44] in the R package Geiger [38]. For this analysis, the entire Hypoptopomatinae phylogeny was divided into 24 lineages to which the total number of species for each lineage (including missing species) was assigned (S3 Table).

### Morphometric data selection, collection and size correction

Both body size and head shape are important correlates of trophic ecological, locomotory and life history characteristics in fishes [45–47]. We therefore measured variation in these traits among Hypoptopomatinae species to provide a morphological index from which to infer ecological diversity.

Maximum standard length (SL in cm) is a useful estimate of body size and associated life-history traits in most fish species [24]. Maximum standard lengths (or maximum body size: MBS) were compiled for all species from original species descriptions, the Check List of the Freshwater Fishes of South and Central America [48], and validated museum records (S2 Table). To quantify head shape, we made 14 point-to-point measurements between external, putatively homologous landmarks using a digital caliper. We collected morphological data for 105 species and 22 genera distributed throughout Hypoptopomatinae (S4 Table). Landmarks and interlandmark distances were a subsample of those originally proposed by Armbruster [49], and were only measured from adult specimens (>18.0 mm SL for Otothyrini, >30 mm SL for Hypoptopomatini, and >50 mm SL for Neoplecostomini).

To minimize allometric (body size) influence on morphometric data, we followed the method of Dryden and Mardia [50] by using the program Past v1.28 [51] to conduct a principal component analysis (PCA), normalize the first two coordinate dimensions, divide all coordinate values by the centroid size for each specimen and conduct a Procrustes superimposition of the left half to a mirrored version of the right half. We then used Past to conduct a PCA on the covariance matrix of phylogenetically corrected residuals.

### Estimating phenotypic diversification rates

To estimate rates of continuous evolution in body size and head shape, we used BAMM v2.1.0 ([41]; see BAMM input values in S2 Table). BAMM was programmed to use two chains running simultaneously for a total of 50 million generations, and to sample tree space every 5,000^th^ generation. MCMC convergence was checked by plotting the output log-likelihood trace using the R package BAMMtools [43] with burn-in set to 0.5. Final BAMM analyses of phenotypic evolution examined 5,000 trees sampled from the posterior distribution. Evolutionary rate dynamics were visualized from BAMM output using BAMMtools.

### Ancestral body size estimation

To estimate rates of body size evolution, we used the contMap function in the R package phytools v0.3–10 [35]. This function maps a continuous character on to a phylogenetic tree by estimating ancestral states at each node using fastAnc (fast estimation of ML ancestral states). Macroevolutionary models were evaluated using the fitContinuous function in the R package geiger [38], which ranks models according to AICc scores.

### Phylomorphospace analysis

To visualize the distribution of modern species and ancestral lineages in morphospace [52], we generated phylomorphospace biplots using the phylomorphospace function in the R package phytools v0.3–10 [35]. The first principal component of size-corrected head shape for each taxonomic grouping was plotted against log maximum body size (MBS). Species were plotted using colors that correspond to their habitat classification.

## Results

### Time-calibrated phylogenetic analysis

We obtained a time-calibrated phylogeny from Roxo et al. [25], which is the most taxonomically comprehensive to date for catfishes of the subfamily Hypoptopomatinae (S1 Fig, *sensu* [26]), with a tree topology that parallels earlier studies (i.e. [25, 27]). The subfamily Hypoptopomatinae is estimated to have originated during the Paleocene, approximately 58.4 Ma (40.8–79.7 Ma 95% highest posterior density, HPD), the tribe Hypoptopomatini and clade composed of Neoplecostomini + Otothyrini during the Lower Eocene, approximately 49.9 Ma (33.6–67.4 Ma 95% HPD), and the tribes Neoplecostomini and Otothyrini during the Lower Eocene, approximately 45.9 Ma (31.0–62.2 Ma 95% HPD). Mean substitution rate for the entire dataset was estimated to be 0.27% substitutions per million years.

### Ancestral habitat estimation

**Fig 1:**
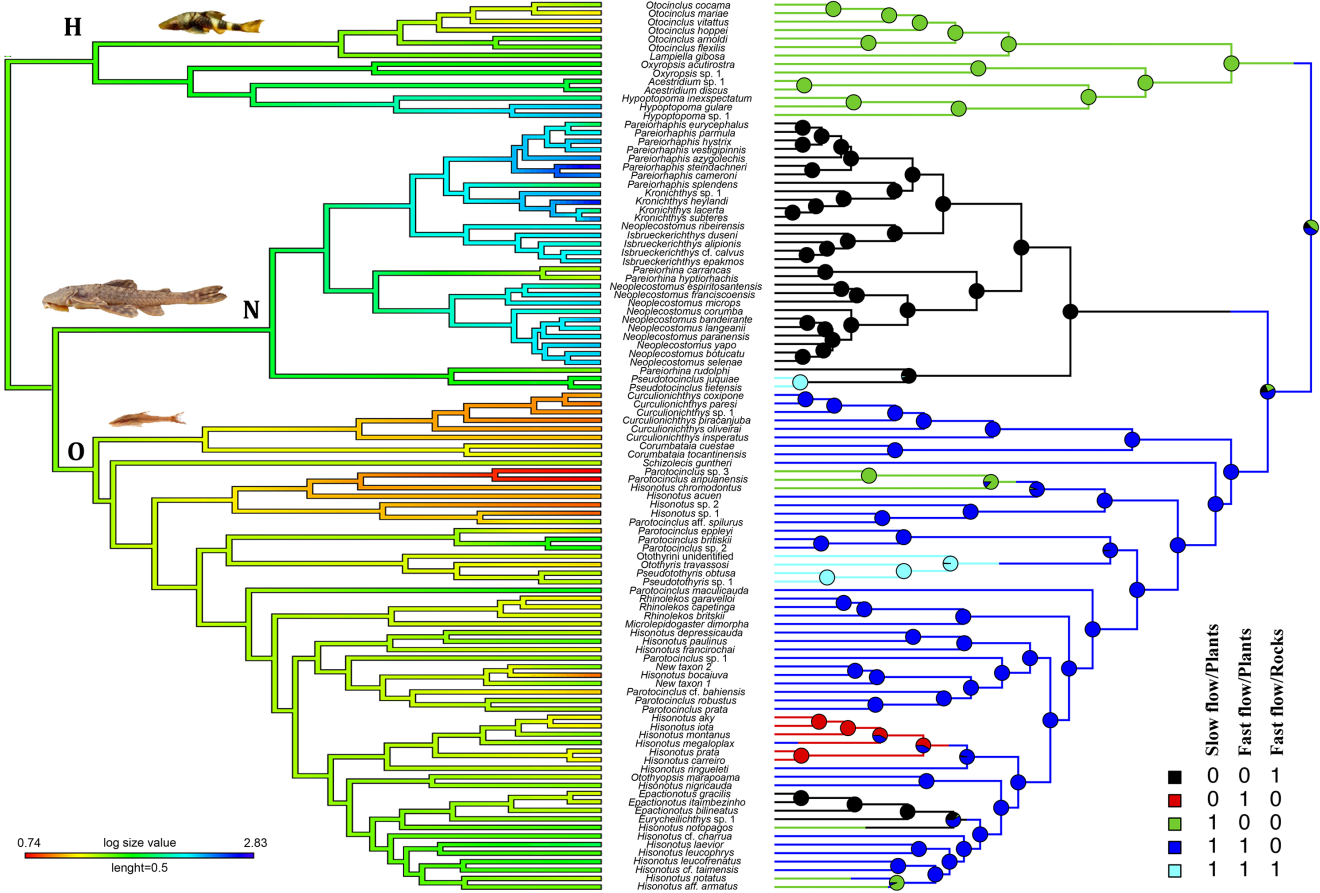
**Left: ContMap cladogram** illustrating estimated ancestral maximum body size (MBS) constructed using a time calibrated phylogeny of Hypoptopomatinae, including the tribes Hypoptopomatini (H), Neoplecostomini (N) and Otothyrini (O). Branch colors correspond to estimates of ln MBS (i.e., maximum standard body length measured from tip of snout to base of caudal fin), with red indicating smaller and green indicating larger MBS. **Right: SIMMAP cladogram** illustrating ancestral habitat estimates for the subfamily Hypoptopomatinae. See S2 Table for habitat classifications of each species.

Our SIMMAP analysis indicated that the tribe Otothyrini occupied all habitat categories, being the tribe occupying the greatest diversity of habitat within subfamily Hypoptopomatinae, whereas the most recent common ancestor (MRCA) of tribe Hypoptopomatini occupied slow-flowing habitat exclusively (Fig 1). In contrast, the entire Neoplecostomini clade, extending back to its MRCA, was found to have occupied fast-flowing rocky habitats almost exclusively (Fig 1). The equal-rates (ER) model best fit our habitat data and was used in the SIMMAP analysis.

### Speciation rate analyses

**Fig 2:**
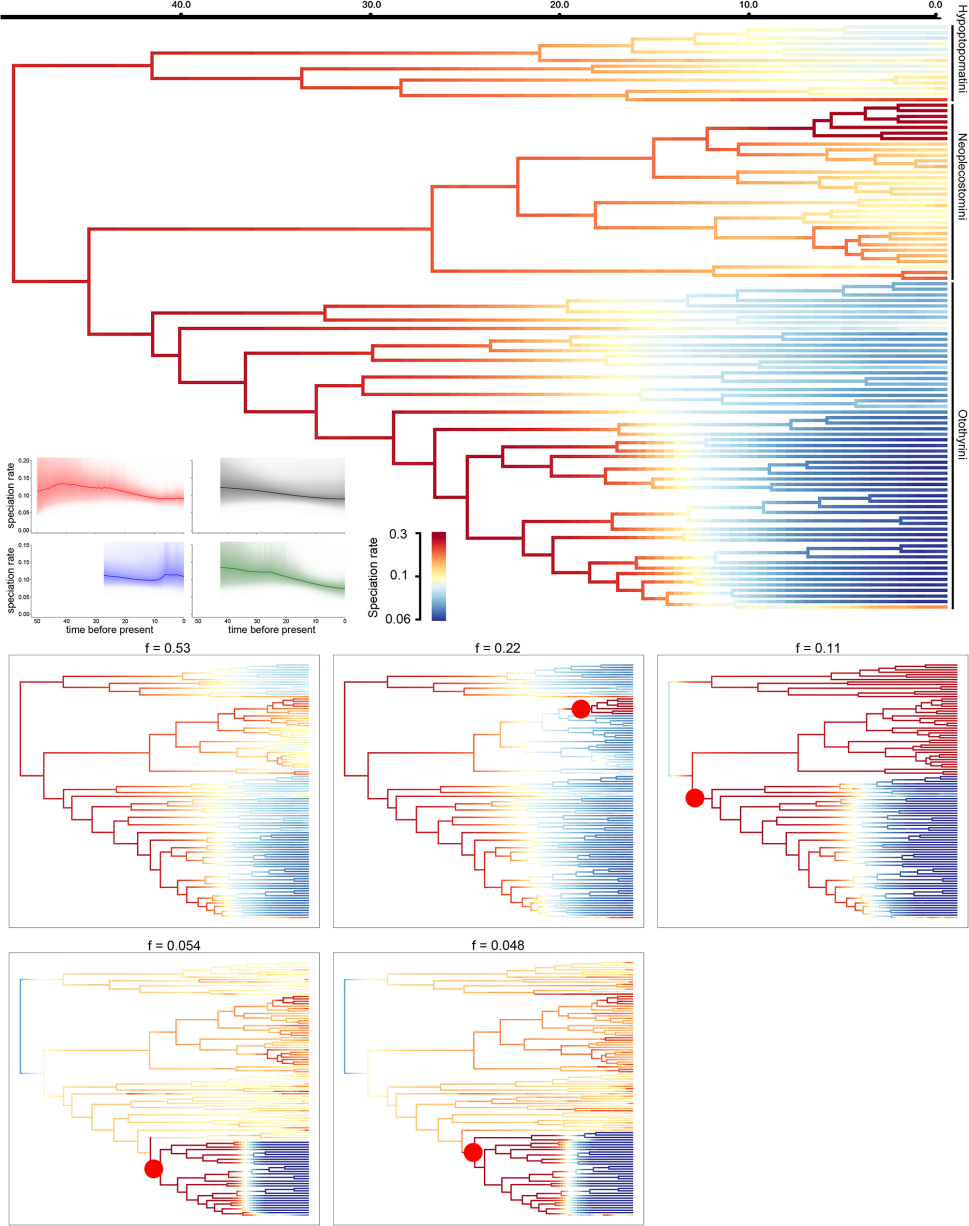
Phylorate plots showing speciation rates for the subfamily Hypoptopomatinae. Branch colors denote instantaneous rates (cool colors = slow, warm = fast). The large cladogram at top depicts mean Phylorate, with colors indicating the mean evolutionary rate across all shift configurations sampled during simulation. Smaller cladograms at bottom show the five distinct shift configurations having the highest posterior probabilities. For each distinct shift configuration, the locations of rate increases are shown as red circles. Text labels denote the posterior probability of each shift configuration. The small Rate-Through-Time plots at left display the cumulative speciation rate from the root of the tree to the present computed from the joint posterior density in BAMM for the entire subfamily (red) and for each tribe (black = Hypoptopomatini; blue = Neoplecostomini; green = Otothyrini). The order of terminal taxa follows that in Fig 1.

**Fig 3:**
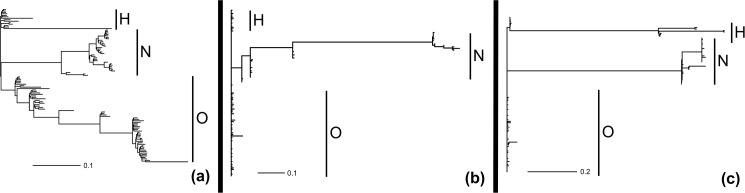
**Marginal probabilities of rate shifts for speciation (a), MBS (b), and head shape (c) in the Hypoptopomatinae tribes Hypoptopomatini (H), Neoplecostomini (N) and Otothyrini (O).** Branch lengths are scaled by the probability that they contain a shift event.

For the subfamily Hypoptopomatinae as a whole, speciation rate increased from the root until approximately 40 Ma, then decreased from that time to the present (Fig 2). Within Hypoptopomatinae, our BAMM analysis indicated an overall deceleration in speciation rate starting with ancestral lineages within the tribes Hypoptopomatini and Otothyrini. Neoplecostomini exhibited a constant overall speciation rate, except for a strong increase along the branch leading to the rapids-dwelling genus *Pareiorhaphis* (Fig 2; Fig 3A). Speciation rate-through-time dynamics for tribes Hypoptopomatini and Otothyrini are also consistent with speciation decreasing from the oldest node to the present, whereas Neoplecostomini showed a small increase in speciation rate near the present (Fig 2).

Our specific test of a speciation rate shift using MEDUSA indicated that speciation rate significantly increased along the branch giving rise to the relatively large-bodied clade comprising the genera *Neoplecostomus*, *Isbrueckerichthys*, *Kronichthys*, *Pareiorhaphis* and two species assigned to the paraphyletic genus *Pareiorhina* (*P*. *carrancas* and *P*. *hyptiorhachis*; S2 Fig).

### Rates of diversification in body size and head shape

**Fig 4:**
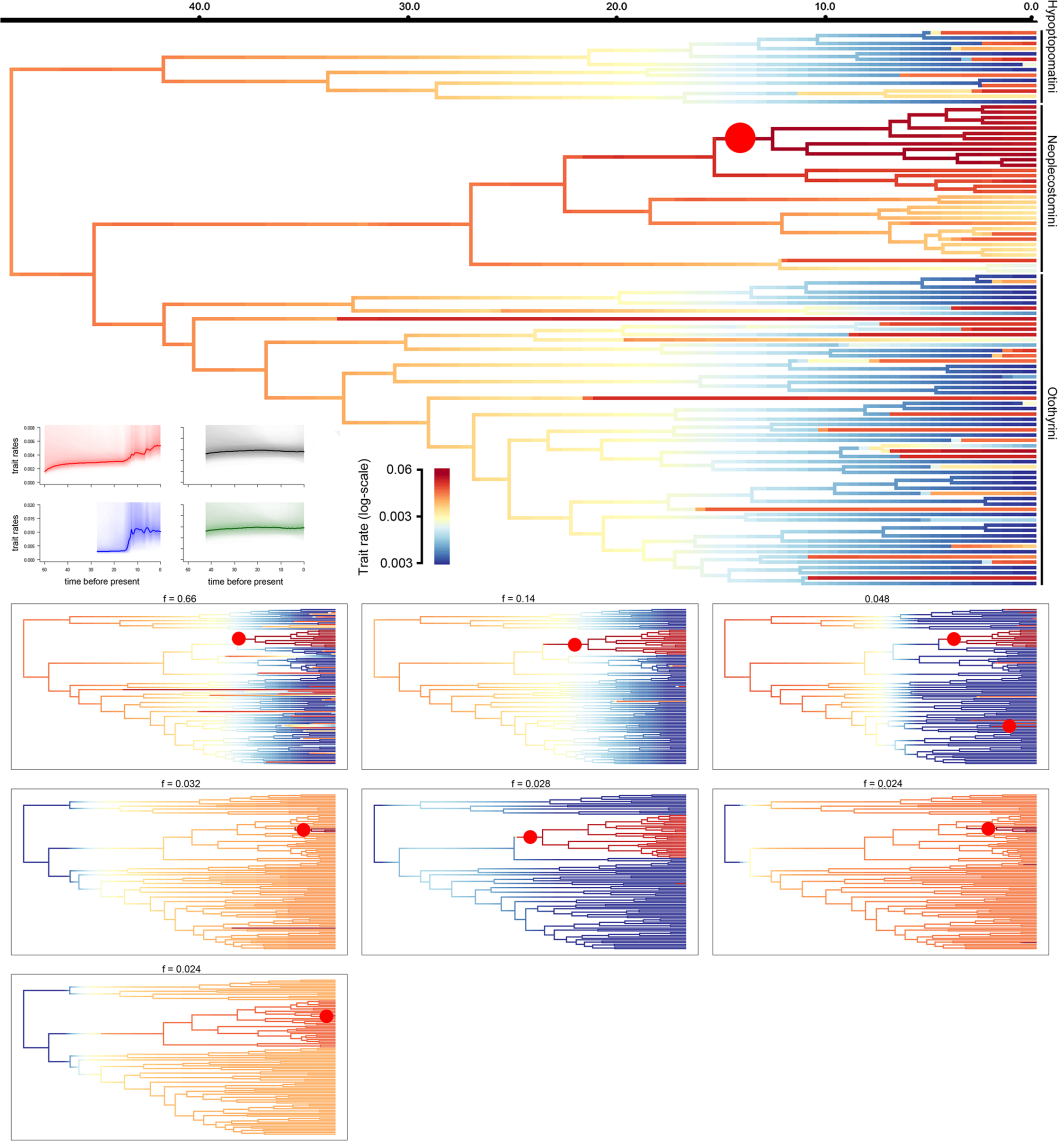
Phylorate plots showing rates of maximum body size (MBS) evolution throughout the subfamily Hypoptopomatinae. Branch colors denote instantaneous rates (cool colors = slow, warm = fast). The large cladogram at top depicts mean Phylorate, with red circles indicating the most frequent rate increases along all sampled trees of the Bayesian analysis. Smaller cladograms at bottom show the seven distinct shift configurations having the highest posterior probabilities. For each distinct shift configuration, the locations of rate increases are shown as red circles. Text labels denote the posterior probability of each shift configuration. The small Rate-Through-Time plots at left display cumulative MBS rates from the root to the present computed from the joint posterior density in BAMM for the subfamily Hypoptopomatinae (red) and each tribe (black = Hypoptopomatini; blue = Neoplecostomini; green = Otothyrini). The order of terminal taxa follows that in Fig 1.

Our BAMM analysis indicated an acceleration in the rate of maximum body size (MBS) evolution within the tribe Neoplecostomini. This was greatest in the clade containing all species of the genera *Pareiorhaphis* and *Kronichthys* (Fig 4). In contrast, MBS diversification was relatively constant in the Hypoptopomatini and decelerated in lineages of the Otothyrini (Fig 4).

**Fig 5:**
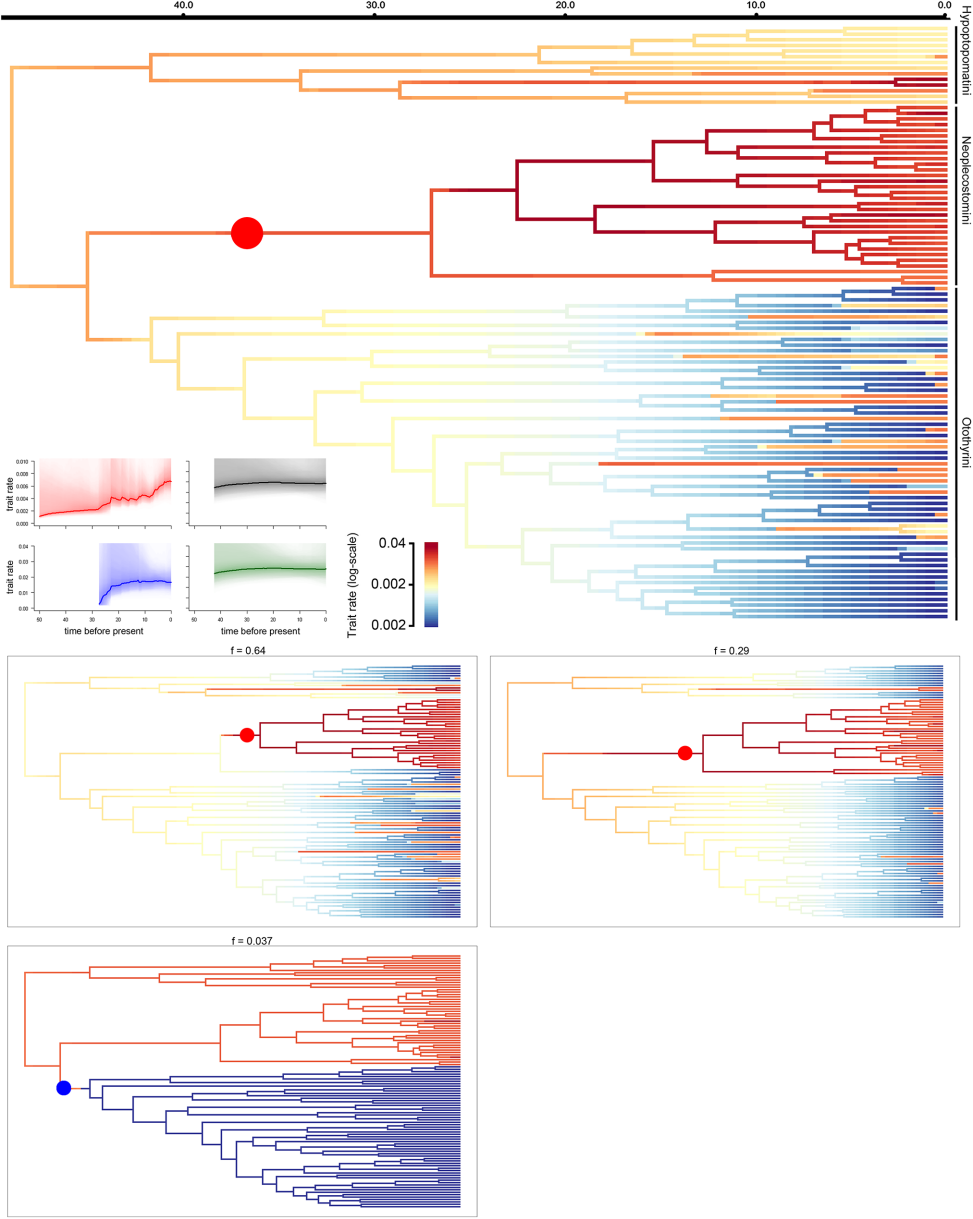
Phylorate plots showing head-shape diversification rates for the subfamily Hypoptopomatinae. Branch colors denote instantaneous rates (cool colors = slow, warm = fast). The large cladogram at top depicts the mean Phylorate with the red circle indicating the most frequent rate increase along all sampled trees of the Bayesian analysis. Smaller cladograms at bottom show the three distinct shift configurations having the highest posterior probabilities for head shape evolutionary rate. For each distinct shift configuration, the locations of rate increases are shown as red circles. Text labels denote the posterior probability of each shift configuration. The small Rate-Through-Time plots at left display the cumulative head-shape diversification rate from the root of the tree to the present computed from the joint posterior density in BAMM for the subfamily Hypoptopomatinae (red plot) and each tribe (black = Hypoptopomatini; blue = Neoplecostomini; green = Otothyrini). The order of terminal taxa follows that in Fig 1.

We also observed an acceleration in head shape diversification along the branch leading to the tribe Neoplecostomini (Fig 5), and evidence of a shift in the rate of head-shape diversification at the node giving rise to Neoplecostomini (Fig 5).

### Estimating ancestral body size

Our ContMAP analysis revealed an evolutionary trend toward larger body sizes in the Neoplecostomini and an overall decrease in body sizes in Otothyrini (Fig 1). Some clades nested within Neoplecostomini showed very fast rates of evolution to both larger (*Kronichthys subteres* and *Pareiorhaphis cameroni*) and smaller (*K*. *lacerta* and *P*. *eurycephalus*) body sizes. The Kappa model best fit our body size data and was used for our ContMAP analysis.

### Phylomorphospace analysis

**Fig 6:**
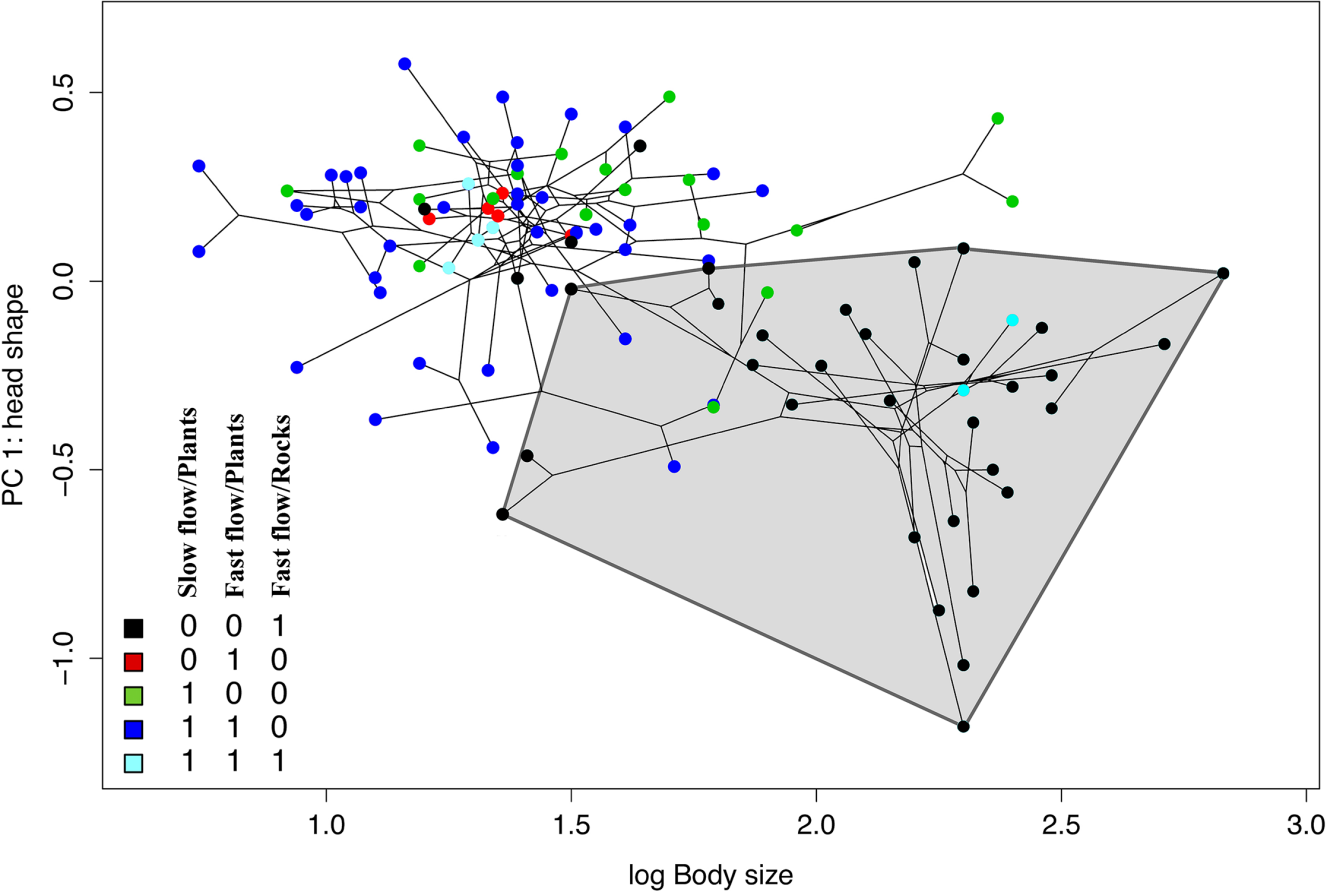
Phylomorphospace plot for the subfamily Hypoptopomatinae in which terminal colors correspond to habitat and the shaded convex hull encloses all examined species of the tribe Neoplecostomini. The y-axis is the first principal component (PC) from a PC analysis of log-normalized linear distances between 14 external landmarks on the head (following head landmarks originally proposed by Armbruster [54]) and the x-axis is log maximum body size (MBS).

The first principal component (PC1) axis of our phylomorphospace analysis explained 52.4% of variation in head shape for all Hypoptopomatinae species. Characters that loaded strongly on PC1 were head–eye length, orbit diameter, snout length, internares width, interorbital width, mouth width, and barbel length (S5 Table). Species of the highly rheophilic tribe Neoplecostomini occupied a distinct region of head shape/body size morphospace (Fig 6), largely exclusive of the morphospace occupied by slower-water dwelling members of the Hypoptopomatini and Otothyrini.

## Discussion

Our analyses indicate that the most recent common ancestor of the cascudinho catfish subfamily Hypoptopomatinae had a relatively small body size (~6 cm SL) and occupied both slow-flowing/vegetated habitat and rapids habitats with fast-flowing water and rocky substrates approximately 58.4 Ma (40.8–79.7 Ma 95% HPD; S1 Fig and Fig 1). Fishes of the tribes Hypoptopomatini and Otothyrini–two of the three major cascudinho subclades–remained largely restricted to small body sizes and similar habitats (Fig 1), and exhibited mostly decreasing or constant rates of speciation (Fig 2) and morphological diversification (Figs 4 and 5). In contrast, the primarily rapids-dwelling tribe Neoplecostomini exhibited constant or increasing rates of speciation and morphological diversification, with a significant shift toward faster speciation in the lineage leading to almost exclusively rapids-dwelling species (S2 Fig). With maximum body sizes ranging from 3.9 to 17.0 cm SL, modern neoplecostomin species exhibit ca. 120% greater size range than the Hypoptopomatini (3.3–14.3 cm SL) and ca. 300% greater size range than the Otothyrini (2.1–6.6 cm SL; S2 Table). Moreover, neoplecostomin species span a broader range of head shape diversity than either of the two other clades combined (Fig 6). Of the three habitat categories occupied by cascudinho catfishes, only the combination of fast-flowing water and rocky substrates appears to have significantly influenced net speciation rates throughout Hypoptopomatinae.

Ecological opportunity is often invoked to explain accelerations in evolutionary diversification following a habitat shift, due to reduced competition associated with the availability of more resources in new habitats [1, 4, 6]. However, the specific role of ecological opportunity is difficult to disentangle from other influences associated with habitat shifts. Such influences may include reduced dispersal ability, the spatial scale of gene flow, and effective population sizes [8, 12, 21], or increases in the complexity or selective strength of adaptive landscapes [9–11, 17]. Contrary to the predictions of ecological opportunity, we predicted that rapids-dwelling Hypoptopomatinae lineages would exhibit accelerated speciation and decelerated morphological diversification. We based these predictions on prior observations or inferences of reductions in body size, dispersal ability, and the spatial scale of gene flow [20–22], and morphological trends suggesting increased stabilizing selection [26] in fast- vs. slow-water specialized fish lineages.

Empirical patterns, however, differed from our predictions. As predicted, speciation increased in Neoplecostomini, but this acceleration was accompanied by larger, not smaller, body sizes. Also, diversification of both body size and head shape significantly increased in neoplecostomin lineages occupying rapids habitats exclusively. These differences between predicted and observed patterns highlight major gaps in our understanding of the evolutionary influence that rapids habitats can have on rheophilic organisms in general, and loricariid catfishs specifically. Questions raised by our study include whether the distinctive and diverse head shapes observed in Neoplecostomini correspond to as yet unrecognized adaptive ecological peaks–such as distinctive benthic food resources (e.g., [53, 54]) or microhabitats–that might only be present in rapids habitats. If this was known, and these niches were underexploited prior to the neoplecostomin invasion of these habitats, then the otherwise poorly supported hypothesis of ecological opportunity would become increasingly plausible. Likewise, it would be valuable to know if the distinctive hydrodynamic environment of rapids habitats selects for a more narrow range of optimal body sizes. If such a size optimum were larger than the Hypoptopomatinae ancestor, but smaller than ancestors of other fish lineages that have shrunk upon invasion of more lotic, faster-flowing habitats (e.g., Gasterosteidae: *Gasterosteus*, [20]; Cichlidae: *Teleocichla*, [47]; Percidae: Etheostomatinae, [55]), the ecological role in divergent body size shifts would be clearer.

Unfortunately, geographic ranges and the spatial scale of gene flow in these species and habitats are unknown, making the job of disentangling evolutionary mechanisms all the more difficult. Studies of relatively recent, intraspecific differentiation between populations or individuals associated with slow- vs. fast-water habitats indicate that the adaptive landscape of these habitats are distinct, but applications of these studies to the Hypoptopomatinae system are limited. For example, Langerhans et al. [56] investigated the effects of different flow rates on body shape in adjacent populations of a mid-water Neotropical tetra (Characidae, *Bryconops caudomaculatus*) and near-bottom dwelling cichlid (Cichlidae, *Biotodoma wavrini*). They observed shifts toward a more fusiform body, higher aspect ratio caudal fin, and respectively upturned (mid-water species) or downturned (near-bottom species) mouths. Likewise, in studies of lake vs. stream populations of three-spine stickleback (*Gasterosteus aculeatus*; [57]), lotic populations were found to have deeper bodies and caudal peduncles. In a review of flow regime studies on fishes, Langerhans [58] found that fishes adapted for life in high-flow environments share several specialized physiological and biomechanical traits related to swimming, including relatively more red muscle, stiffer bodies, higher steady swimming performance, and lower unsteady swimming performance.

Hypoptopomatinae species, however, are entirely benthic, have dorsoventrally depressed bodies, entirely ventral mouths and oral disks, and rarely swim freely in the water column, preferring instead to make short movements between substrate attachment sites (authors’ pers. obs.). Indeed, the oral disk of all members of the family Loricariidae seems to facilitate attachment and station-holding in fast-water habitats without otherwise specialized body morphologies or enhanced swimming performance [59]. Similar morphological specializations have repeatedly evolved in a wide range of other fast-water specialized fish lineages throughout the world [17]. In a recent study of trait-based fish community structure in a large Neotropical river rapid, Fitzgerald et al. [60] found that loricariid catfishes were a dominant component of the rapid’s fish community, clustered around a distinctive portion of trait space, and were evenly spaced within their region of trait space. Thus, multiple lines of evidence suggest that the ancestral body plan shared by all loricariids predisposes this group to successfully occupy rapids habitats, and that coexistence among diverse loricariid assemblages requires relatively minor variations on this theme. Our results and those of Fitzgerald et al. [60] highlight the need for a better understanding of these variable traits and their ecological correlates.

Despite the elusiveness of a single compelling narrative to explain the observed diversification rate shifts in fast- vs. slow-water specialized Hypoptopomatinae lineages, results of this study should serve as robust support for the further investigation and conservation of riverine rapids habitats. These habitats can be challenging for fishes to occupy, and to investigate in real-time, because of the strong hydraulic forces that define them. Macroevolutionary studies such as this provide an alternative to working directly in the habitat by focusing instead on specimens that have been removed from it. There is an urgent need, however, for studies such as this to be complemented with detailed functional, ecological and population genetic data so that a more complete understanding of eco-evolutionary dynamics in these distinctive but threatened ecosystems can be achieved. Given the intensity and global scale of especially tropical river rapids development [61], such data should be gathered soon.

## Supporting information

S1 Fig Time calibrated tree of Hypoptopomatinae from Roxo et al. [30] used in the present study. All nodes have a Bayesian posterior probability higher than 0.95. Duplicate terminals were deleted from the original time calibrated tree of Roxo et al. [30]. See S1 Table for all taxa information.
(TIF)

S2 Fig Diversity tree from our MEDUSA analysis of lineage diversification in the subfamily Hypoptopomatinae. Clades are collapsed to represent stem lineages and colored by extant species diversity. Clades with unusual diversification rates are denoted with numbers: 1 (yellow) denotes a significant lineage diversification rate increase compared with the background (Bg) in large-bodied species of the tribe Neoplecostomini, and 2 (blue) indicates a significant lineage diversification rate decrease in the lineage leading to the genus *Schizolecis*. Estimates for net diversification rate (r) and relative extinction rate (e) are included in the upper left table. See S4 Table for taxonomic divisions and species richnesses.
(TIF)

S1 Table Species included in the present study. ANSP = Academy of Natural Sciences of Drexel University, Philadelphia; AUM = Auburn University Natural History Museum; LBP = Laboratório de Biologia e Genética de Peixes, Universidade Estadual Paulista; MCP = Museu de Ciências e Tecnologia, Pontifícia Universidade Católica do Rio Grande do Sul; MNRJ = Museu Nacional da Universidade Federal do Rio de Janeiro; NUP = Núcleo de Pesquisas em Limnologia, Ictiologia e Aqüicultura, Universidade Estadual de Maringá; MHNG = Museum of Natural History of the City of Geneva.
(DOCX)

S2 Table Species of the ingroup included in the present study with information about habitat, maximum body size in centimeter and in log (MBS), genera division used in BAMM, percent of species sampled from a lineage used in BAMM, and head shape values also used in BAMM. Abbreviation in the table are: MBS–maximum body size values in log used in BAMM analysis; Gen-Div–Genera division used in BAMM; Perc–percent of sampled species of the lineage in BAMM; HS–head shape values used in BAMM analysis. The habitat classification follows Crampton (2011).
(DOCX)

S3 Table Subdivision of lineages of Hypoptopomatinae and number of species included in each line used for MEDUSA analysis. The order of taxa follows the same present in S2 Fig.
(DOCX)

S4 Table Head morphological characters. (XLSX)

S5 Table Variable loadings in the first Principal Component Analysis (PCA 1) for head shape of combined samples of Hypoptopomatinae tribes. (DOCX)
