# Supplementary figures and images for: Shift from slow- to fast-water habitats accelerates lineage and phenotype evolution in a clade of Neotropical suckermouth catfishes (Loricariidae: Hypoptopomatinae)
Source: PLoS One. 2017 Jun 7;12(6):e0178240. doi: 10.1371/journal.pone.0178240 (PMC5462362; doi:10.1371/journal.pone.0178240)

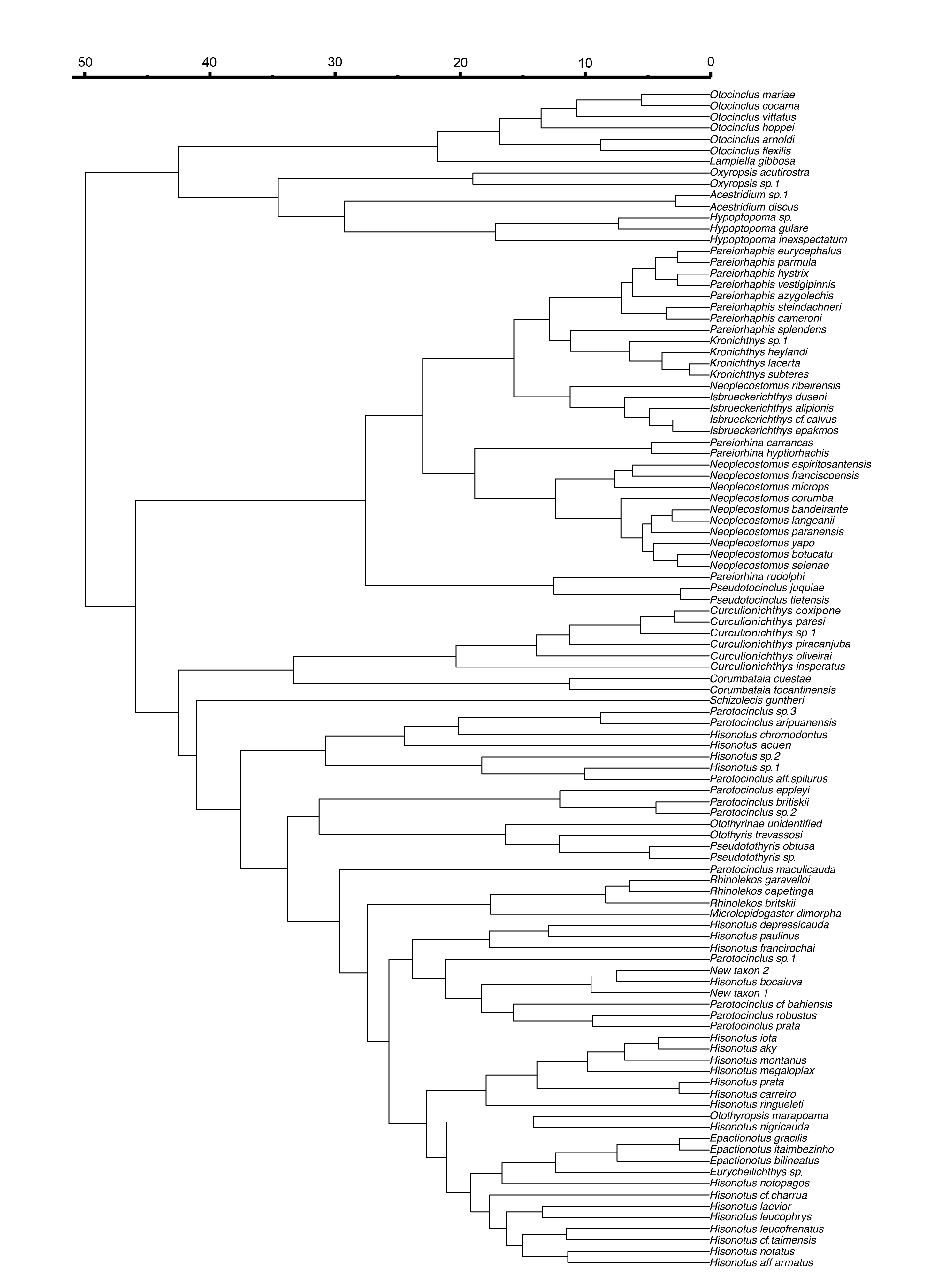

Supplement: S1 Fig — All nodes have a Bayesian posterior probability higher than 0.95. Duplicate terminals were deleted from the original time calibrated tree of Roxo et al. [30]. See S1 Table for all taxa information. (TIF) [file pone.0178240.s001.tif]

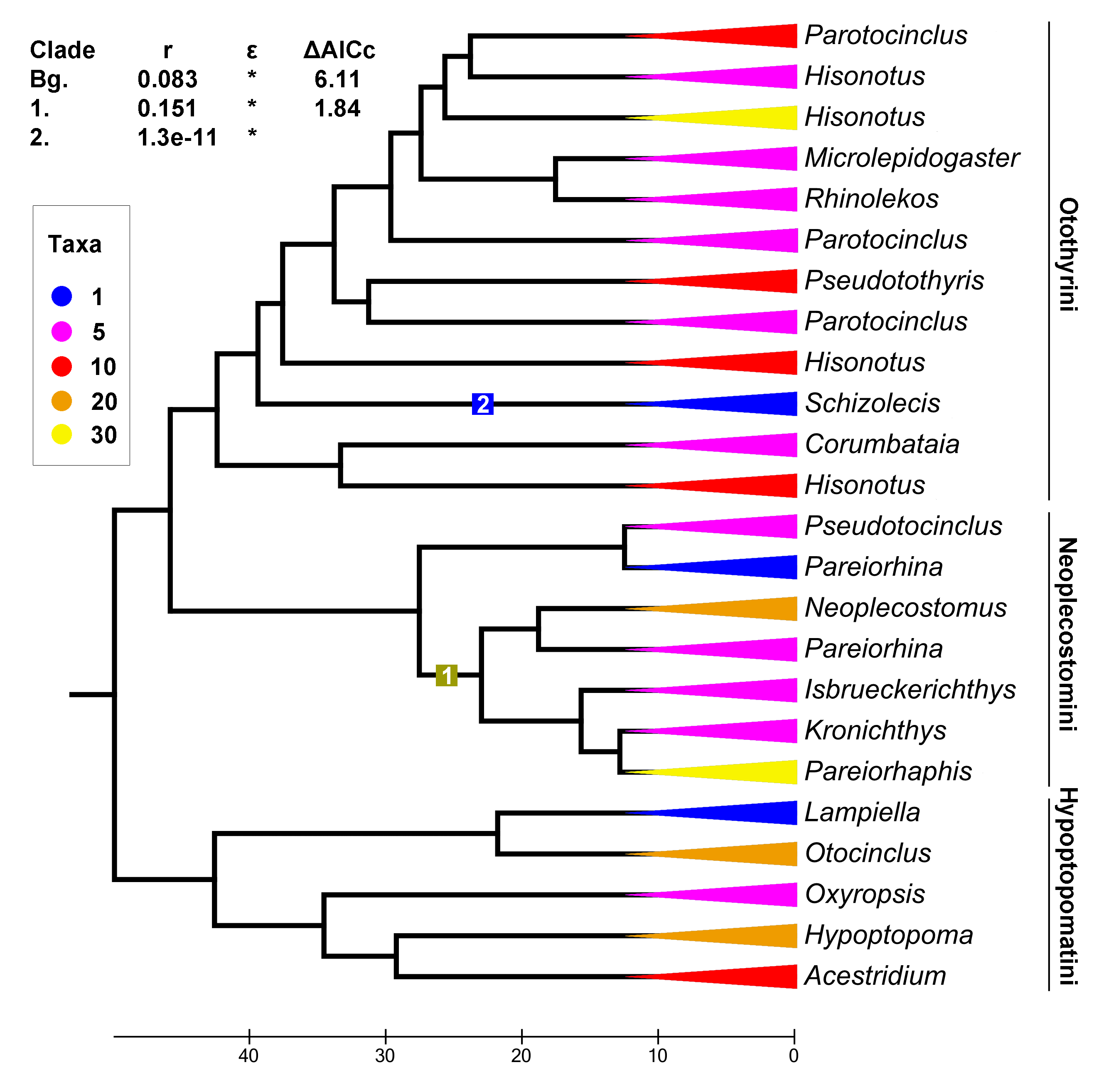

Supplement: S2 Fig — Clades are collapsed to represent stem lineages and colored by extant species diversity. Clades with unusual diversification rates are denoted with numbers: 1 (yellow) denotes a significant lineage diversification rate increase compared with the background (Bg) in large-bodied species of the tribe Neoplecostomini, and 2 (blue) indicates a significant lineage diversification rate decrease in the lineage leading to the genus Schizolecis. Estimates for net diversification rate (r) and relative extinction rate (e) are included in the upper left table. See S4 Table for taxonomic divisions and species richnesses. (TIF) [file pone.0178240.s002.tif]
