# Supplementary material for: Shift from slow- to fast-water habitats accelerates lineage and phenotype evolution in a clade of Neotropical suckermouth catfishes (Loricariidae: Hypoptopomatinae)
Source: PLoS One. 2017 Jun 7;12(6):e0178240. doi: 10.1371/journal.pone.0178240 (PMC5462362; doi:10.1371/journal.pone.0178240)
Supplement: S2 Table — Abbreviation in the table are: MBS–maximum body size values in log used in BAMM analysis; Gen-Div–Genera division used in BAMM; Perc–percent of sampled species of the lineage in BAMM; HS–head shape values used in BAMM analysis. The habitat classification follows Crampton (2011). (DOCX) [file pone.0178240.s004.docx]

**Supplementary Table 2.** Species of the ingroup included in the present study with information about habitat, maximum body size in centimeter and in log (MBS), genera division used in BAMM, percent of species sampled from a lineage used in BAMM, and head shape values also used in BAMM. Abbreviation in the table are: MBS – maximum body size values in log used in BAMM analysis; Gen-Div – Genera division used in BAMM; Perc – percent of sampled species of the lineage in BAMM; HS – head shape values used in BAMM analysis. The habitat classification follows Crampton (2011).

| Number | Collection No | Fish No | Species | Slow flow/Plants | Fast flow/Plants | Fast flow/Rocks | Size (cm) | MBS | Gen-Div | Perc | HS |
| --- | --- | --- | --- | --- | --- | --- | --- | --- | --- | --- | --- |
| 1 | LBP 6037 | 29054 | *Hisonotus* aff. *armatus* | 1 | 0 | 0 | 4.60 | 1.53 | 1 | 0.72 | 0.17648 |
| 2 | LBP 3472 | 20258 | *Hisonotus notatus* | 1 | 0 | 0 | 4.00 | 1.39 | 1 | 0.72 | 0.22732 |
| 3 | LBP 4765 | 25554 | *Hisonotus* cf. *taimensis* | 1 | 1 | 0 | 5.00 | 1.61 | 1 | 0.72 | 0.40867 |
| 4 | LBP 7407 | 35655 | *Hisonotus leucofrenatus* | 1 | 1 | 0 | 6.00 | 1.79 | 1 | 0.72 | 0.28438 |
| 5 | MCP 41351 |  | *Hisonotus leucophrys* | 1 | 1 | 0 | 5.00 | 1.61 | 1 | 0.72 | 0.083766 |
| 6 | LBP 3376 | 21246 | *Hisonotus laevior* | 1 | 1 | 0 | 6.60 | 1.89 | 1 | 0.72 | 0.23925 |
| 7 | LBP 4720 | 24941 | *Hisonotus* cf. *charrua* | 1 | 1 | 0 | 5.05 | 1.62 | 1 | 0.72 | 0.14842 |
| 8 | MCP 40762 |  | *Hisonotus notopagos* | 1 | 0 | 0 | 5.17 | 1.64 | 1 | 0.72 | 0.35781 |
| 9 | LBP 4723 | 24951 | *Eurycheilichthys* sp. 1 | 0 | 0 | 1 | 4.50 | 1.50 | 1 | 0.72 | 0.10581 |
| 10 | LBP 4871 | 24919 | *Epactionotus bilineatus* | 0 | 0 | 1 | 4.00 | 1.39 | 1 | 0.72 | 0.0067382 |
| 11 | MCP 23683 |  | *Epactionotus itaimbezinho* | 0 | 0 | 1 | 3.80 | 1.34 | 1 | 0.72 | 0.21832 |
| 12 | MCP 23606 |  | *Epactionotus gracilis* | 0 | 0 | 1 | 3.90 | 1.36 | 1 | 0.72 | 0.23299 |
| 13 | LBP 3335 | 20465 | *Hisonotus nigricauda* | 1 | 1 | 0 | 5.00 | 1.50 | 1 | 0.72 | 0.44262 |
| 14 | LBP 5528 | 25343 | *Otothyropsis marapoama* | 1 | 1 | 0 | 3.85 | 1.35 | 1 | 0.72 | 0.17244 |
| 15 | LBP 4863 | 24930 | *Hisonotus ringueleti* | 1 | 1 | 0 | 3.90 | 1.36 | 1 | 0.72 | 0.48822 |
| 16 | MCP 40943 |  | *Hisonotus carreiro* | 0 | 1 | 0 | 3.77 | 1.33 | 1 | 0.72 | 0.19254 |
| 17 | MCP 40942 |  | *Hisonotus prata* | 0 | 1 | 0 | 3.32 | 1.20 | 1 | 0.72 | 0.19027 |
| 18 | MCP 41352 |  | *Hisonotus megaloplax* | 1 | 1 | 0 | 4.71 | 1.55 | 1 | 0.72 | 0.13791 |
| 19 | LBP 13055 | 51035 | *Hisonotus montanus* | 0 | 1 | 0 | 4.50 | 1.50 | 1 | 0.72 | 0.12027 |
| 20 | MCP 41474 |  | *Hisonotus aky* | 0 | 1 | 0 | 3.45 | 1.24 | 1 | 0.72 | 0.19518 |
| 21 | MCP 40029 |  | *Hisonotus iota* | 0 | 1 | 0 | 3.34 | 1.21 | 1 | 0.72 | 0.16487 |
| 22 | LBP 8249 | 38464 | *Parotocinclus prata* | 1 | 1 | 0 | 4.19 | 1.43 | 2 | 1.00 | 0.12992 |
| 23 | LBP 8258 | 38298 | *Parotocinclus robustus* | 1 | 1 | 0 | 4.20 | 1.44 | 2 | 1.00 | 0.2216 |
| 24 | LBP 7182 | 34694 | *Parotocinclus* cf. *bahiensis* | 1 | 1 | 0 | 3.10 | 1.13 | 2 | 1.00 | 0.093163 |
| 25 | LBP 5867 | 28345 | *New taxon* 1 | 1 | 1 | 0 | 5.00 | 1.51 | 2 | 1.00 | 0.12996 |
| 26 | LBP 17402 | 67143 | *Hisonotus bocaiuva* | 1 | 1 | 0 | 2.56 | 0.94 | 2 | 1.00 | -0.22919 |
| 27 | LBP 7244 | 33302 | *New taxon* 2 | 1 | 1 | 0 | 5.00 | 1.51 | 2 | 1.00 | 0.12666 |
| 28 | LBP 3457 | 20293 | *Parotocinclus* sp. 1 | 1 | 1 | 0 | 4.30 | 1.46 | 2 | 1.00 | -0.024727 |
| 29 | LBP 13923 | 58218 | *Hisonotus francirochai* | 1 | 1 | 0 | 3.60 | 1.28 | 2 | 1.00 | 0.38179 |
| 30 | LBP 3932 | 22661 | *Hisonotus paulinus* | 1 | 1 | 0 | 4.00 | 1.39 | 2 | 1.00 | 0.30589 |
| 31 | LBP 2910 | 18756 | *Hisonotus depressicauda* | 1 | 1 | 0 | 5.00 | 1.61 | 2 | 1.00 | -0.15324 |
| 32 | LBP 4854 | 25626 | *Microlepidogaster dimorpha* | 1 | 1 | 0 | 3.77 | 1.33 | 3 | 0.44 | -0.2367 |
| 33 | LBP 7245 | 34405 | *Rhinolekos britskii* | 1 | 1 | 0 | 3.83 | 1.34 | 3 | 0.44 | 0.14186 |
| 34 | LBP 5848 | 28365 | *Rhinolekos* sp. 1 | 1 | 1 | 0 | 3.50 | 1.25 | 3 | 0.44 | 0.034833 |
| 35 | LBP 7246 | 33305 | *Rhinolekos garavelloi* | 1 | 1 | 0 | 3.62 | 1.29 | 3 | 0.44 | 0.2575 |
| 36 | LBP 2869 | 18571 | *Parotocinclus maculicauda* | 1 | 1 | 0 | 6.00 | 1.78 | 4 | 0.33 | 0.053122 |
| 37 | LBP 4722 | 24946 | *Pseudotothyris* sp. 1 | 1 | 1 | 1 | 4.00 | 1.39 | 5 | 0.57 | 0.20421 |
| 38 | LBP 2094 | 13851 | *Pseudotothyris obtusa* | 1 | 1 | 1 | 4.00 | 1.39 | 5 | 0.57 | 0.367 |
| 39 | LBP 1971 | 13685 | *Otothyris travassosi* | 1 | 1 | 1 | 3.20 | 1.16 | 5 | 0.57 | 0.57609 |
| 40 | MHNG 2586.95 | BR1200 | *Otothyrinae unidentified* | 1 | 1 | 1 | 5.00 | 1.31 | 5 | 0.57 | 0.10852 |
| 41 | LBP 6950 | 35328 | *Parotocinclus* sp. 2 | 1 | 1 | 0 | 6.10 | 1.71 | 6 | 0.50 | -0.49205 |
| 42 | ANSP 179131 |  | *Parotocinclus britskii* | 1 | 1 | 0 | 6.00 | 1.79 | 6 | 0.50 | -0.32776 |
| 43 | AUM 43947 |  | *Parotocinclus eppleyi* | 1 | 1 | 0 | 3.00 | 1.10 | 6 | 0.50 | -0.36725 |
| 44 | LBP 5624 | 35327 | *Parotocinclus* aff. *spilurus* | 1 | 1 | 0 | 4.00 | 1.39 | 7 | 0.58 | 0.28468 |
| 45 | LBP 334 | 4276 | *Hisonotus* sp. 1 | 1 | 1 | 0 | 2.51 | 0.92 | 7 | 0.58 | 0.2392 |
| 46 | LBP 8276 | 38487 | *Hisonotus* sp. 2 | 1 | 1 | 0 | 2.51 | 0.92 | 7 | 0.58 | 0.2392 |
| 47 | LBP 16277 | 61771 | *Hisonotus acuen* | 1 | 1 | 0 | 2.91 | 1.07 | 7 | 0.58 | 0.28676 |
| 48 | LBP 7948 | 35749 | *Hisonotus chromodontus* | 1 | 0 | 0 | 3.03 | 1.11 | 7 | 0.58 | -0.030663 |
| 49 | LBP 10981 | 50459 | *Parotocinclus aripuanensis* | 1 | 0 | 0 | 2.10 | 0.74 | 7 | 0.58 | 0.078385 |
| 50 | LBP 2414 | 16276 | *Parotocinclus* sp. 3 | 1 | 0 | 0 | 2.10 | 0.74 | 7 | 0.58 | 0.30574 |
| 51 | LBP 2988 | 19646 | *Schizolecis guntheri* | 1 | 1 | 0 | 4.00 | 1.39 | 8 | 1.00 | 0.23107 |
| 52 | LBP 1653 | 11477 | *Corumbataia tocantinensis* | 1 | 1 | 0 | 3.80 | 1.34 | 9 | 0.80 | -0.44116 |
| 53 | LBP 2001 | 12191 | *Corumbataia cuestae* | 1 | 1 | 0 | 3.30 | 1.19 | 9 | 0.80 | -0.21798 |
| 54 | LBP 5529 | 25381 | *Curculionichthys insperatus* | 1 | 1 | 0 | 3.00 | 1.10 | 9 | 0.80 | 0.0090896 |
| 55 | LBP 1325 | 11238 | *Curculionichthys oliveirai* | 1 | 1 | 0 | 2.84 | 1.04 | 9 | 0.80 | 0.27738 |
| 56 | LBP 17256 | 66655 | *Curculionichthys piracanjuba* | 1 | 1 | 0 | 2.56 | 0.94 | 9 | 0.80 | 0.20075 |
| 57 | MNRJ 29502 | 490 | *Curculionichthys* sp. 1 | 1 | 1 | 0 | 2.75 | 1.01 | 9 | 0.80 | 0.28103 |
| 58 | LBP 17532 | 68706 | *Curculionichthys paresi* | 1 | 1 | 0 | 2.62 | 0.96 | 9 | 0.80 | 0.17691 |
| 59 | LBP 5062 | 26057 | *Curculionichthys coxipone* | 1 | 1 | 0 | 2.91 | 1.07 | 9 | 0.80 | 0.19673 |
| 60 | LBP 2931 | 18994 | *Pseudotocinclus tietensis* | 1 | 1 | 1 | 6.00 | 1.80 | 10 | 0.75 | -0.060191 |
| 61 | LBP 616 | 7564 | *Pseudotocinclus juquiae* | 1 | 1 | 1 | 5.87 | 1.78 | 10 | 0.75 | 0.033934 |
| 62 | LBP 4391 | 24189 | *Pareiorhina rudolphi* | 0 | 0 | 1 | 4.50 | 1.50 | 10 | 0.75 | -0.021072 |
| 63 | LBP 7383 | 34843 | *Neoplecostomus selenae* | 0 | 0 | 1 | 10.17 | 2.32 | 11 | 0.76 | -0.82235 |
| 64 | LBP 7525 | 34832 | *Neoplecostomus botucatu* | 0 | 0 | 1 | 10.20 | 2.32 | 11 | 0.76 | -0.37478 |
| 65 | NUP 3560 | 9701 | *Neoplecostomus yapo* | 0 | 0 | 1 | 10.60 | 2.36 | 11 | 0.76 | -0.49997 |
| 66 | LBP 2732 | 17444 | *Neoplecostomus paranensis* | 0 | 0 | 1 | 9.82 | 2.28 | 11 | 0.76 | -0.63604 |
| 67 | LBP 5901 | 27990 | *Neoplecostomus langeanii* | 0 | 0 | 1 | 8.55 | 2.15 | 11 | 0.76 | -0.31678 |
| 68 | LBP 2861 | 18616 | *Neoplecostomus bandeirante* | 0 | 0 | 1 | 10.90 | 2.39 | 11 | 0.76 | -0.56024 |
| 69 | NUP 2528 | 9423 | *Neoplecostomus corumba* | 0 | 0 | 1 | 7.83 | 2.06 | 11 | 0.76 | -0.076065 |
| 70 | LBP 645 | 7593 | *Neoplecostomus microps* | 0 | 0 | 1 | 10.00 | 2.30 | 11 | 0.76 | 0.086719 |
| 71 | LBP 6537 | 31681 | *Neoplecostomus franciscoensis* | 0 | 0 | 1 | 7.50 | 2.01 | 11 | 0.76 | -0.22454 |
| 72 | LBP 2551 | 15243 | *Neoplecostomus espiritosantensis* | 0 | 0 | 1 | 10.00 | 2.30 | 11 | 0.76 | -1.1807 |
| 73 | LBP 8380 | 37559 | *Pareiorhina hyptiorhachis* | 0 | 0 | 1 | 3.88 | 1.36 | 12 | 0.50 | -0.61813 |
| 74 | LBP 8368 | 37565 | *Pareiorhina carrancas* | 0 | 0 | 1 | 4.10 | 1.41 | 12 | 0.50 | -0.46219 |
| 75 | LBP7385 | 34852 | *Isbrueckerichthys epakmos* | 0 | 0 | 1 | 10.00 | 2.30 | 13 | 1.00 | -0.20789 |
| 76 | LBP 6389 | 29765 | *Isbrueckerichthys* cf. *calvus* | 0 | 0 | 1 | 9.00 | 2.20 | 14 | 1.00 | 0.050339 |
| 77 | LBP 7373 | 34853 | *Isbrueckerichthys alipionis* | 0 | 0 | 1 | 8.20 | 2.10 | 14 | 1.00 | -0.14043 |
| 78 | LBP 2650 | 17402 | *Isbrueckerichthys duseni* | 0 | 0 | 1 | 10.00 | 2.30 | 14 | 1.00 | -1.018 |
| 79 | LBP 7384 | 34837 | *Neoplecostomus ribeirensis* | 0 | 0 | 1 | 9.00 | 2.20 | 14 | 1.00 | -0.67915 |
| 80 | LBP 515 | 6334 | *Kronichthys subteres* | 0 | 0 | 1 | 12.00 | 2.48 | 15 | 1.00 | -0.24954 |
| 81 | LBP 795 | 8304 | *Kronichthys lacerta* | 0 | 0 | 1 | 7.00 | 1.95 | 15 | 1.00 | -0.32732 |
| 82 | LBP 2122 | 15096 | *Kronichthys heylandi* | 0 | 0 | 1 | 15.00 | 2.71 | 15 | 1.00 | -0.16689 |
| 83 | LBP 1766 | 12886 | *Kronichthys* sp. 1 | 0 | 0 | 1 | 11.00 | 2.40 | 15 | 1.00 | -0.27992 |
| 84 | LBP 748 | 8257 | *Pareiorhaphis splendens* | 0 | 0 | 1 | 6.50 | 1.87 | 15 | 1.00 | -0.22233 |
| 85 | MCP 41275 |  | *Pareiorhaphis cameroni* | 0 | 0 | 1 | 17.00 | 2.83 | 16 | 0.33 | 0.020547 |
| 86 | LBP 902 | 7989 | *Pareiorhaphis steindachneri* | 0 | 0 | 1 | 12.00 | 2.48 | 16 | 0.33 | -0.3372 |
| 87 | MCP 41909 |  | *Pareiorhaphis azygolechis* | 0 | 0 | 1 | 11.70 | 2.46 | 16 | 0.33 | -0.12389 |
| 88 | LBP 1161 | 8935 | *Pareiorhaphis vestigipinnis* | 0 | 0 | 1 | 10.00 | 2.30 | 16 | 0.33 | -0.28948 |
| 89 | LBP 701 | 7363 | *Pareiorhaphis hystrix* | 0 | 0 | 1 | 11.00 | 2.40 | 16 | 0.33 | -0.10346 |
| 90 | MCP 41747 |  | *Pareiorhaphis parmula* | 0 | 0 | 1 | 9.45 | 2.25 | 16 | 0.33 | -0.8728 |
| 91 | MCP 41458 |  | *Pareiorhaphis eurycephalus* | 0 | 0 | 1 | 6.30 | 1.89 | 16 | 0.33 | -0.14376 |
| 92 | LBP 4042 | 22905 | *Hypoptopoma inexspectatum* | 1 | 0 | 0 | 14.30 | 1.96 | 17 | 0.20 | 0.13446 |
| 93 | LBP 3081 | 19713 | *Hypoptopoma gulare* | 1 | 0 | 0 | 11.00 | 2.40 | 17 | 0.20 | 0.21128 |
| 94 | MHNG 2709.024 | MUS 388 | *Hypoptopoma* sp. 1 | 1 | 0 | 0 | 10.70 | 2.37 | 17 | 0.20 | 0.43124 |
| 95 | LBP 3165 | 19315 | *Acestridium discus* | 1 | 0 | 0 | 6.70 | 1.90 | 18 | 0.28 | -0.030523 |
| 96 | LBP 7204 | 35332 | *Acestridium* sp. 1 | 1 | 0 | 0 | 6.00 | 1.79 | 18 | 0.28 | -0.33458 |
| 97 | LBP 6973 | 35324 | *Oxyropsis* sp. 1 | 1 | 0 | 0 | 5.87 | 1.77 | 19 | 0.66 | 0.15033 |
| 98 | LBP 4300 | 23945 | *Oxyropsis acutirostra* | 1 | 0 | 0 | 5.70 | 1.74 | 19 | 0.66 | 0.26852 |
| 99 | LBP 2652 | 17407 | *Lampiella gibbosa* | 1 | 0 | 0 | 5.00 | 1.61 | 20 | 0.36 | 0.24239 |
| 100 | LBP 877 | 8564 | *Otocinclus flexilis* | 1 | 0 | 0 | 5.50 | 1.70 | 20 | 0.36 | 0.4887 |
| 101 | MCP 25234 |  | *Otocinclus arnoldi* | 1 | 0 | 0 | 4.80 | 1.57 | 20 | 0.36 | 0.29607 |
| 102 | LBP 5310 | 26831 | *Otocinclus hoppei* | 1 | 0 | 0 | 3.30 | 1.19 | 20 | 0.36 | 0.039753 |
| 103 | LBP 5132 | 26233 | *Otocinclus vittatus* | 1 | 0 | 0 | 3.30 | 1.19 | 20 | 0.36 | 0.21689 |
| 104 | MCP 34842 |  | *Otocinclus cocama* | 1 | 0 | 0 | 4.40 | 1.48 | 20 | 0.36 | 0.33721 |
| 105 | MHNG no number | SU07-350 | *Otocinclus mariae* | 1 | 0 | 0 | 3.30 | 1.19 | ̶ | ̶ | 0.35918 |
