# Supplementary material for: Shift from slow- to fast-water habitats accelerates lineage and phenotype evolution in a clade of Neotropical suckermouth catfishes (Loricariidae: Hypoptopomatinae)
Source: PLoS One. 2017 Jun 7;12(6):e0178240. doi: 10.1371/journal.pone.0178240 (PMC5462362; doi:10.1371/journal.pone.0178240)
Supplement: S3 Table — The order of taxa follows the same present in S2 Fig. (DOCX) [file pone.0178240.s005.docx]

**Supplementary Table 3.** Subdivision of lineages of Hypoptopomatinae and number of species included in each line used for MEDUSA analysis. The order of taxa follows the same present in Fig. S2.

|  |  | Taxonomical richness for each lineage |
| --- | --- | --- |
| 1 | *Parotocinclus* | 7 |
| 2 | *Hisonotus* | 3 |
| 3 | *Hisonotus* | 29 |
| 4 | *Microlepidogaster* | 5 |
| 5 | *Rhinolekos* | 3 |
| 6 | *Parotocinclus* | 3 |
| 7 | *Pseudotothyris* | 6 |
| 8 | *Parotocinclus* | 3 |
| 9 | *Curculionichthys* | 7 |
| 10 | *Schizolecis* | 1 |
| 11 | *Corumbataia* | 4 |
| 12 | *Hisonotus* | 6 |
| 13 | *Pseudotocinclus* | 3 |
| 14 | *Pareiorhina* | 1 |
| 15 | *Neoplecostomus* | 13 |
| 16 | *Pareiorhina* | 4 |
| 17 | *Isbrueckerichthys* | 5 |
| 18 | *Kronichthys* | 3 |
| 19 | *Pareiorhaphis* | 21 |
| 20 | *Lampiella* | 1 |
| 21 | *Otocinclus* | 18 |
| 22 | *Oxyropsis* | 3 |
| 23 | *Hypoptopoma* | 15 |
| 24 | *Acestridium* | 7 |
