# Supplementary material for: Shift from slow- to fast-water habitats accelerates lineage and phenotype evolution in a clade of Neotropical suckermouth catfishes (Loricariidae: Hypoptopomatinae)
Source: PLoS One. 2017 Jun 7;12(6):e0178240. doi: 10.1371/journal.pone.0178240 (PMC5462362; doi:10.1371/journal.pone.0178240)
Supplement: S5 Table — (DOCX) [file pone.0178240.s007.docx]

**Supplementary Table 5.** Variable loadings in the first Principal Component Analysis (PCA 1) for head shape of combined samples of Hypoptopomatinae tribes.

|  |  | PCA1 (head shape) |
| --- | --- | --- |
| 1 | Head length | 0.01858 |
| 2 | Cleithral width | -0.01287 |
| 3 | Head-pectoral length | -0.00124 |
| 4 | Head-eye length | 0.1134 |
| 5 | Orbit diameter | 0.2724 |
| 6 | Snout length | -0.1068 |
| 7 | Internares width | 0.1457 |
| 8 | Interorbital width | 0.1658 |
| 9 | Head depth | 0.0423 |
| 10 | Mouth length | -0.03791 |
| 11 | Mouth width | -0.2039 |
| 12 | Barbel length | 0.89 |
| 13 | Dentary tooth cup length | -0.0781 |
| 14 | Premaxillary tooth cup length | -0.09653 |
